# Meteorological Observations for January, 1857, Atlanta, Ga.

**Published:** 1857-02

**Authors:** J. G. Westmoreland


### METEOROLOGICAL OBSERVATIONS FOR JANUARY, 1857, AT ATLANTA, GA.

| JANUARY. | TEMPERATURE. | | | WIND. | REMARKS. |
|---|---|---|---|---|---|
| | 7 A. M. | 2 P. M. | 7 P. M. | | |
| 1 | 35 | 42 | 36 | E. | Cloudy. |
| 2 | 34 | 36 | 35 | E. | Cloudy—Rain 1¾ inch. |
| 3 | 34 | 41 | 29 | N. W. | Fair—Windy. |
| 4 | 22 | 41 | 30 | N. W. | Fair. |
| 5 | 28 | 52 | 38 | N. | Light clouds. |
| 6 | 34 | 45 | 34 | N. W. | Fair. |
| 7 | 29 | 42 | 29 | N. W. | Cloudy—Rain 3-16 inch. |
| 8 | 24 | 32 | 26 | N. W. | Light clouds. |
| 9 | 24 | 40 | 28 | W. | Fair. |
| 10 | 36 | 40 | 30 | N. | Cloudy—Rain ¾ inch. |
| 11 | 22 | 30 | 24 | N. W. | Fair. |
| 12 | 20 | 35 | 32 | W. | Fair. |
| 13 | 30 | 40 | 30 | W. | Fair. |
| 14 | 27 | 50 | 43 | W. | Fair. |
| 15 | 26 | 36 | 24 | N. W. | Fair. |
| 16 | 28 | 38 | 34 | N. E. | Cloudy—Drizzily. |
| 17 | 36 | 42 | 41 | S. W. | Cloudy—Rain ½ inch. |
| 18 | 18 | 14 | 12 | N. W. | Snow 1½ inch. |
| 19 | 2 | 18 | 14 | N. W. | Fair. |
| 20 | 12 | 32 | 32 | E. | Cloudy—Light Snow. |
| 21 | 32 | 35 | 16 | N. W. | Fair. |
| 22 | 6 | 20 | 18 | N. W. | Fair. |
| 23 | 12 | 28 | 20 | N. W. | Snow ½ inch. |
| 24 | 16 | 35 | 30 | N. | Cloudy. |
| 25 | 30 | 42 | 34 | N. W. | Fair. |
| 26 | 34 | 44 | 36 | N. E. | Light clouds. |
| 27 | 36 | 54 | 38 | E. | Light clouds. |
| 28 | 42 | 54 | 50 | S. E. | Cloudy—Rain ¼ inch. |
| 29 | 48 | 50 | 44 | N. E. | Cloudy—Rain ½ inch. |
| 30 | 38 | 38 | 35 | E. | Cloudy—Rain ¼ inch. |
| 31 | 34 | 40 | 38 | W. | Fair. |

Furnished by

J. G. WESTMORELAND, M. D.

